# Immunogenic Cell Death: An Emerging Target in Gastrointestinal Cancers

**DOI:** 10.3390/cells11193033

**Published:** 2022-09-28

**Authors:** Marta Chiaravalli, Alexia Spring, Antonio Agostini, Geny Piro, Carmine Carbone, Giampaolo Tortora

**Affiliations:** 1 Medical Oncology, Fondazione Policlinico Universitario Agostino Gemelli, IRCCS, 00168 Rome, Italy; 2 Medical Oncology, Università Cattolica del Sacro Cuore, 00168 Rome, Italy

**Keywords:** colorectal cancer, DAMPs, gastric cancer, gastrointestinal cancers, hepatocellular carcinoma, HMGB-1, immunogenic cell death, pancreatic cancer

## Abstract

Immunogenic cell death (ICD) is a regulated form of cell death that induces the activation of both innate and adaptive immune responses through the release of damage-associated molecular patterns (DAMPs) and their subsequent recognition by pattern-recognition receptors (PRRs), generating specific CD8+ T lymphocytes. Thus, ICD inducers (such as certain chemotherapeutic agents, targeted therapies, radiation, and oncolytic viruses) could become a potential cancer treatment by providing antitumour immunity and cancer vaccination. Moreover, their combination with immunotherapy, especially with immune checkpoint inhibitors, could overcome the immunosuppressive tumour microenvironment that characterises certain cancers, including gastrointestinal cancers. This review will provide insights into the role of ICD induction in colorectal, gastric, pancreatic, and hepatocellular carcinomas. Specifically, we will discuss the main mechanisms involved in ICD, their potential application in gastrointestinal cancer treatment, and the latest clinical trial updates.

## 1. Introduction

For a long time, apoptosis has been considered a tolerogenic type of cell death, in contrast to necrosis, its pro-inflammatory counterpart. Nowadays, it is well-established that apoptosis of infected or malignant cells can activate both innate and adaptive immune responses through the release of damage-associated molecular patterns (DAMPs). DAMPs release can initiate a molecular cascade that leads to the generation of tumour-specific CD8+ T lymphocytes and immunological memory: this is called immunogenic cell death (ICD) [1]. 

**Figure 1:**
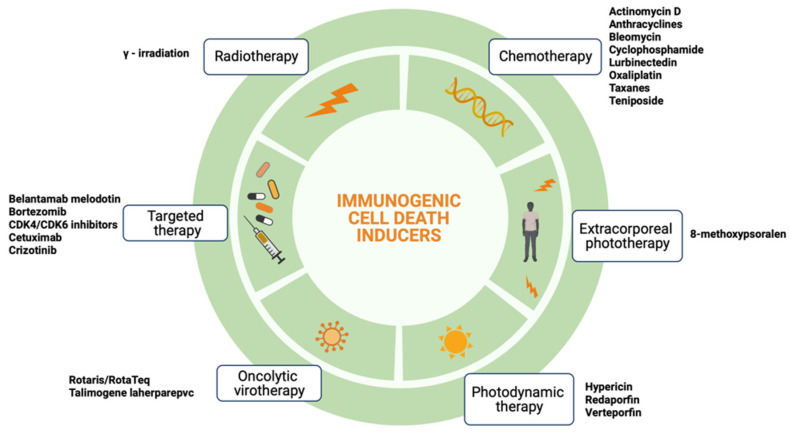
Immunogenic cell death inducers. Immunogenic cell death inducers can be grouped into six different categories: chemotherapy agents, radiotherapy, extracorporeal phototherapy, photodynamic therapy, oncolytic virotherapy, and targeted therapy.

ICD seems to be a promising approach in cancer therapy; nevertheless, only a few oncological therapeutic agents are known to induce ICD, including anthracyclines, oxaliplatin, cetuximab, bortezomib, radiotherapy, oncolytic virotherapy, photodynamic therapy, and extracorporeal photochemotherapy [2,3,4,5,6,7,8,9]; a complete list is provided in Figure 1. These agents promote alterations either at the cell surface or in the extracellular microenvironment compatible with the induction of ICD (Table 1).

One of the main hallmarks of cancer, described by Hanahan and Weinberg in 2011, is the avoidance of immune destruction [10], which can be achieved by secreting immunosuppressive factors, recruiting immunosuppressive cells as T regulatory cells, and downregulating the activation of cytotoxic lymphocytes. In this context, the use of immune checkpoint inhibitors (ICIs) has changed the natural history of several solid malignancies, including melanoma [11,12,13], lung [14,15], and genitourinary cancers [16,17,18]. Indeed, both cancer cells and antigen-presenting cells (APCs) express on their plasma membrane ligands able to bind to immune checkpoints on lymphocytes, suppressing their activation; ICIs interfere with this mechanism, restoring T-cell activity [19].

Little is known about the interaction of ICIs with ICD inducers and their potential role in stimulating a durable immune response, which could ultimately provide cancer vaccination, especially in cancers where immunotherapy alone shows limited results, such as gastrointestinal (GI) cancers [20]. GI cancers represent a significant challenge as they account for about 26% of the global cancer incidence and, in 2018, were responsible for 3.4 million cancer deaths [21]. Herein, we review the therapeutic strategies that could boost ICD in GI malignancies, their potential synergistic effect with other agents, such as ICIs, their different outcomes based on genomic profiling, and the future perspectives in this research field.

## 2. Immunogenic Cell Death

ICD is a type of regulated cell death that occurs in infected and malignant cells and elicits antigen-specific immune response inducing immunological memory. The role of ICD became more relevant with the evolution from unicellular to multicellular organisms, protecting the host from potential danger [22].

The immunogenicity of cancer cells depends on the production of tumour neoantigens, which are not included in the repertoire of central or peripheral tolerances, and oncofetal antigens, normally not expressed in adult tissues, where their peptides are presented on major histocompatibility complex (MHC) class I and II molecules [23]. Nevertheless, antigen presentation to T-cells requires co-stimulatory signals provided by APCs and dendritic cells (DCs) to be efficient, otherwise resulting in anergy and peripheral tolerance; this is called adjuvanticity and represents an essential step [24]. 

ICD involves the release of “eat me” and “find me” signals, known as DAMPs, from dying cells that are recognised by pattern-recognition receptors (PRRs) expressed by DCs, finally inducing their maturation and subsequent efficient CD8+ T-cell cross-priming.

ICD-related DAMPs comprise various molecules; the main “eat me” signal is represented by the endoplasmic reticulum chaperon calreticulin (CALR) exposure on the plasma membrane, which interacts with LDL receptor-related protein 1 (LRP1) on DCs [25].

Other factors favouring APCs recruitment are the autophagy-dependent secretion of adenosine triphosphate (ATP), which binds to the purinergic receptor P2Y2 (P2YR2) on DC precursors [26], and the passive liberation of the cytosolic protein annexin A1 (ANXA1), which guides DCs to dying cells and facilitates their physical interaction [27]. Antigen cross-presentation to CD8+ lymphocytes can be stimulated by the nuclear release of high-mobility group box 1 (HMGB-1), which binds to Toll-like receptor 4 (TLR4) on DCs [28].

Furthermore, mitochondrial deoxyribonucleic acid (DNA) cytosolic accumulation promotes the activation of cyclic GMP-AMP synthase (CGAS) and the stimulation of interferon response cGMP interactor 1 (STING 1), with the subsequent release of IFN-1 and other cytokines that support the initiation of adaptive immunity [29].

In addition, the cell surface exposure of heat-shock proteins (HSPs), including HSP70 and HSP90, which normally behave as molecular chaperones for other proteins, is involved in ICD [30].

**Figure 2:**
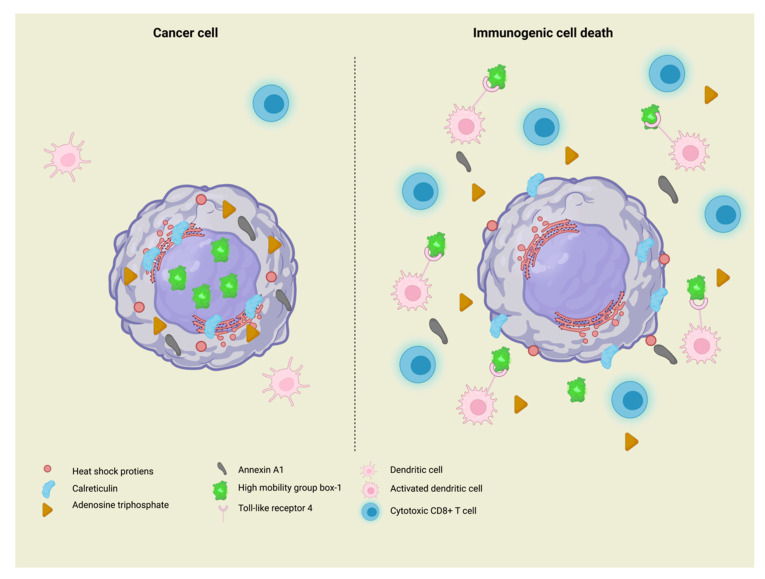
Schematic representation of immunogenic cell death (ICD). Cancer cells undergoing ICD release damage-associated molecular patterns. In particular, they expose the endoplasmic reticulum protein calreticulin and heat shock proteins on the plasma membrane and release cytosolic adenosine triphosphate, cytosolic annexin A1 protein, and nuclear high-mobility group box-1 (HMGB-1) in the extracellular space. Moreover, dendritic cells become activated and, due to the expression of Toll-like receptor 4, bind to the HMGB-1, now in the extracellular space. This results in the recruitment of cytotoxic CD8+ cells to the tumour site.

A further ICD hallmark is the phosphorylation of eukaryotic translation initiation factor 2 subunit α (eIF2α), which correlates with CALR exposure and tumour infiltration by DCs and CD8+ T-cells; this suggests that dampening microbial (especially viral) messenger ribonucleic acid (mRNA) translation could have been one of the first mechanisms of response to infectious pathogens [22,31]. A schematic representation of the ICD and the involved DAMPs release is shown in Figure 2.

Notably, the capacity of ICD to activate an efficient immune response is limited by tumour microenvironment (TME) components, such as infiltration by CD4+ CD25+ FOXP3+ regulatory T-cells (Tregs), which promote immunosuppression through cytotoxic T-lymphocyte-associated protein 4 (CTLA-4) expression and interleukin-10 (IL-10) release, and tumour fibrotic response, which is particularly significant in pancreatic ductal adenocarcinomas (PDACs) and alters-primed T-cell lymphocytes mobility [32,33]. 

In conclusion, ICD is a type of regulated cell death that promotes the activation of innate and adaptive immune responses and immunological memory in infectious and malignant diseases. These features involving the host’s immune response could be strategic for cancer therapy effectiveness.

## 3. ICD in Colorectal Cancer

Oxaliplatin is part of the chemotherapy backbone for colorectal cancer (CRC). More than ten years ago, Tesniere et al. demonstrated its role as an ICD inducer through pre-apoptotic CALR exposure on the plasma membrane and post-apoptotic HMGB-1 release [3]. Moreover, they investigated the effect of a *TLR4* loss-of-function allele (*Asp299Gly*) on clinical outcomes in oxaliplatin-treated metastatic CRC patients, and the results showed increased progression-free survival (PFS) and overall survival (OS) in wild-type *TLR4* patients.

In the first-line setting, the choice between anti-angiogenic and anti-epidermal growth factor receptor (EGFR) agents is driven by RAS and BRAF mutational status and tumour sidedness; in contrast, no predictive factors have been identified to favour oxaliplatin over irinotecan or vice versa. Given this premise, Arai et al. [34] investigated whether single nucleotide polymorphisms (SNPs) in ICD-related genes could influence clinical outcomes in oxaliplatin-treated patients. This retrospective analysis demonstrated an association between *ANXA1 rs1050305* and OS (A/A vs. any G, HR = 1.87, 95% CI 1.04–3.35) and *LRP1 rs1799986* and OS (C/C vs. any T, HR = 1.69, 95% CI 1.07–2.70), proving that genetic variants in the ICD pathway could affect oxaliplatin-based therapy efficacy. However, further prospective studies are required to validate these results.

As few chemotherapeutic agents are known to induce ICD, new drugs have been investigated in CRC, such as bullatacin, isolated from the fruit of *Annona atemoya,* and lipotecan, a novel topoisomerase I inhibitor. Huang et al. demonstrated that the latter could elicit surface exposure of CALR and enhance the efficacy of neoadjuvant chemoradiotherapy (CRT) in BALB/c mice inoculated with CT26 mouse colon carcinoma cells; in fact, the complete response rate in the lipotecan-based CRT group was higher than that of the 5-fluorouracil-based CRT group (33.3 vs. 16.7%, respectively). These findings suggest that lipotecan could become part of neoadjuvant CRT in CRC and improve clinical outcomes in a setting in which recurrence rates are still relevant (5–15% at 5 years) [35,36].

Immunotherapy with ICIs has recently shown significant results in deficient mismatch repair (dMMR)/microsatellite instability (MSI)-high CRCs, which represent a limited subset of all cases (3–5%). In fact, the KEYNOTE 177 trial demonstrated an increased PFS with pembrolizumab in untreated dMMR/MSI-high metastatic CRCs (PFS 16.5 vs. 8.2 months with standard therapy, HR 0.59), with fewer treatment-related adverse events compared to chemotherapy [37]. Notably, in the final analysis, there was no significant difference in OS between the two treatment groups [38]. The efficacy of ICIs in dMMR/MSI-high patients is due in part to the high burden of neoantigens arising from the hypermutated state of cancer cells, which triggers an immune response. In contrast, AtezoTRIBE is a phase II clinical trial that investigated the efficacy of the combination of atezolizumab with first-line FOLFOXIRI (5-fluorouracil, oxaliplatin, irinotecan, and leucovorin) and bevacizumab in metastatic CRCs, irrespective of microsatellite status [39]. Concerning the proficient MMR (pMMR)/microsatellite stable (MSS) population, which accounts for more than 90% of cases, the underlying rationale is that the induction of ICD by oxaliplatin combined with the ability of bevacizumab to restore adaptive immune mechanisms, normally downregulated by vascular endothelial growth factor (VEGF), could amplify T-cell-mediated cancer cell killing caused by ICIs. The primary endpoint of the study, PFS in the intention-to-treat population, was met (13.1 vs. 11.5 months, HR 0.69, 80% CI 0.56–0.85). Further investigations of this combination regimen in pMMR/MSS metastatic colon cancer are warranted, especially in patients with high immunoscore IC (a synthetic measure of CD8+ T lymphocyte infiltration, programmed death ligand 1 (PD-L1) cell abundance, and the proximity between PD-L1 cells and CD8+ T-cells) or high tumour mutational burden.

The addition of ICIs to chemoradiotherapy for locally advanced rectal cancer was investigated according to the same principles. In this setting, radiotherapy, a well-known ICD inducer, can overcome the resistance of pMMR CRCs to ICIs. In particular, the AVANA phase II clinical trial enrolled 101 patients, of whom 23% achieved a complete pathological response and 61.5% a major pathological response, thus suggesting promising activity of chemoradiotherapy and avelumab in the neoadjuvant setting [40]. 

Conversely, the CHINOREC phase II trial, presented at the American Society of clinical oncology (ASCO) 2022 meeting, evaluated neoadjuvant chemoradiotherapy, sequential ipilimumab, and nivolumab in the same population. The interim analysis demonstrated that the aforementioned treatment schedule did not increase the surgical reoperation rates. Further data and prospective phase III trials are needed to validate this therapeutic approach in CRC patients [41].

## 4. ICD in Gastric Cancer

Since CRT represents a pre-operative treatment option for gastric cancer (GC), Petersen et al. [42] investigated the ability of 5-fluorouracil and X-ray irradiation to stimulate ICD in seven GC cell lines. After demonstrating that CRT could improve CALR exposure on the plasma membrane, a known ICD marker, they discovered that PD-L1 and Galectin-9 (Gal-9) expression was enhanced. PD-L1 and Gal-9 are immunosuppressive ligands which play their part by binding, respectively, to PD-1 and T-cell immunoglobulin and mucin-domain containing 3 (Tim-3), both present on immune cells’ plasma membrane. Therefore, they focused on two representative cell lines (MKN7 and MKN74) and investigated whether PD-L1 blockade could overcome immune evasion. They finally demonstrated that PD-L1 blocking antibodies caused better DCs maturation in the coculture setting.

These results not only confirm the role of Food and Drug Administration (FDA)-approved pembrolizumab and nivolumab in GC therapy [43,44] but also provide preclinical evidence about the efficacy of CRT combined with ICIs.

Since it has been demonstrated that neoadjuvant chemotherapy with oxaliplatin-containing regimens can alter the infiltration and subtypes of immune cells by significantly decreasing FOXP3+ Treg cells and increasing CD8+ cytotoxic T-cells and TCR diversity in GCs, several ongoing studies are evaluating the efficacy of combination therapy with ICIs [45]. 

The phase II PANDA trial investigated the effect of neoadjuvant atezolizumab plus docetaxel, oxaliplatin, and capecitabine (DOC) on tumour response rate in GCs [46]; a major pathological response (MPR) was observed in 14 out of 20 patients (70%; 95% CI 46–88%), including nine pathological complete responses (pCR) (45%; 95% CI 52–96%), which appeared to be higher than in historical controls (pCR 16% and MPR 37% with neoadjuvant FLOT (5-fluorouracil, oxaliplatin, docetaxel, and leucovorin)). Of note, at a median follow-up of 29 months, none of the patients with an MPR experienced disease recurrence, suggesting that such data need to be validated in a phase III randomised, controlled trial.

In an analogue phase IIb trial (DANTE) comparing perioperative FLOT and atezolizumab in combination with FLOT, the interim analysis demonstrated that downsizing favoured the latter combination regimen (pT0: 15 vs. 23%; pN0: 54 vs. 68%), especially in GCs with a higher PD-L1 expression; further data are awaited [47]. 

Second-line treatment efficacy in GCs is limited, and new therapeutic options are urgently needed in this setting. Tougeron et al. [48] investigated the FOLFIRI regimen (5-fluorouracil, irinotecan, and leucovorin) in combination with durvalumab alone or durvalumab plus tremelimuamb as a second-line option. The latter combination regimen demonstrated clinically relevant efficacy (mPFS of 6 months) with an acceptable safety profile, paving the way for phase III clinical trials. 

## 5. ICD in Pancreatic Cancer

Pancreatic ductal adenocarcinoma (PDAC) is well known for its dismal prognosis; its poor response to chemotherapy, which nowadays is the only approved treatment; and its unresponsiveness to immunotherapy. Indeed, it is characterised by an immunosuppressive TME and a strong desmoplastic reaction, which not only mechanically interferes with drug delivery at the tumour site but also plays an important role in tumour homeostasis and aggressiveness, with major crosstalk with pancreatic cancer cells (PCC) [49]. In this scenario, ICD induction could convert the immunosuppressive phenotype of this cancer into an immune “active” environment.

The first-line treatment for metastatic PDAC includes regimens based on oxaliplatin, a known ICD inducer, or gemcitabine, a non-ICD inducer. Zhao et al. investigated whether the amphiphilic diblock copolymer nanoparticles encapsulated form (NP) of gemcitabine and oxaliplatin could enhance ICD [50]. Both NP-gemcitabine and NP-oxaliplatin induced significantly more apoptosis than their free counterparts. Moreover, both the release of HMGB-1 and the secretion of ATP were remarkably increased after treatment with NP-oxaliplatin compared to oxaliplatin; this was not confirmed for NP-gemcitabine or gemcitabine treatment. Although the role of HMGB-1 in PDAC is debated [51], as it has been associated with tumour progression and poor therapy response [52], this study evaluated its role as a DAMP and its ability to induce an immune response. These results indicate that NP encapsulation of the drug could increase DAMPs release with chemotherapeutic agents that are known ICD inducers; however, this has not been confirmed for non-ICD inducers. Moreover, they found that the NP encapsulation enhanced DCs’ maturation which, as mentioned before, is another important step in ICD induction. In order to prove that these effects were related to the encapsulation of ICD inducers, both immunodeficient and immunocompetent mice were treated with different formulations: treatment with both NP-oxaliplatin and oxaliplatin was more efficient in immunocompetent than in immunodeficient mice. In particular, animals treated with NP-oxaliplatin had an increased rate of ICD compared to those treated with oxaliplatin (24.6% vs. 10%); this difference was not evident in NP-gemcitabine versus gemcitabine-treated animals. Additionally, CD8+ cytotoxic T-cells were more abundant in the TME of PDAC treated with NP-oxaliplatin than in those treated with oxaliplatin. Further studies should focus on combinatorial therapy with immune checkpoint inhibitors and NP-ICD inducers, such as NP-oxaliplatin.

Following the same rationale, nanocarrier particles of encapsulated oxaliplatin, together with an IDO inhibitor (IND) prodrug, were injected subcutaneously, locally, or systemically in animal models of PDAC. IDO inhibitors are used as a method to reverse the immunosuppressive effect of regionally expressed indoleamine 2,3-dioxygenase 1 (IDO1) at the tumour site, which usually impedes T-cells maturation by interfering with the kynurenine pathway. Subcutaneous vaccination with oxaliplatin generated a systemic immune response, and the local injection of oxaliplatin combined with IND-PL nanoparticles induced the recruitment of CD8+ cytotoxic T-cells and depletion of regulatory Foxp3+ T-cells in the TME. Remarkably, the combined delivery of oxaliplatin and IND-PL into a nanocarrier achieved a synergistic effect, inducing a potent immune response against PDAC cells, thus resulting in an increased OS in mouse models. This approach seems promising, even though no current translation into clinical practice has been reported [53].

It is interesting to note that even if gemcitabine does not induce ICD, it still has some immunomodulatory properties. A study by Smith et al. evaluated gemcitabine as a monotherapy or in combination with other agents (pomalidomide, oxaliplatin, or zoledronic acid): gemcitabine was the only agent capable of upregulating the expression of human leukocyte antigen (HLA)-class I, CD47, and PD-L1 in cancer cells at concentrations with minimal cytotoxicity [54]. This is interesting because low-dose gemcitabine could be combined with other treatments in future studies, exploiting its immunogenic ability. However, gemcitabine was unable to induce the expression of ICD markers, confirming that it is not an ICD-inducer.

A recent randomised phase II trial (NCT03214250; PRINCE trial) evaluated the addition of nivolumab and/or sotigalimab, a CD40 agonistic antibody, to first-line gemcitabine plus nab-paclitaxel, with the aim of overcoming the immunosuppressive features of PDAC and inducing immunologic memory [55]. The primary endpoint of 1-year OS was reached in the nivolumab plus chemotherapy group (57.7%, *p* = 0.006 compared to the historical 35%) but not in the other arms. An exploratory analysis showed that the presence of pretreatment CD4+ T-cells was predictive of increased survival in patients treated with nivolumab plus gemcitabine and nab-paclitaxel, which could be used as a prospective biomarker for future studies. 

As previously mentioned, both chemotherapy and radiotherapy are known to induce ICD. Murine models of locally advanced PDAC were treated with concurrent stereotactic body radiotherapy (SBRT) and FOLFIRINOX (5-fluorouracil, irinotecan, oxaliplatin, and leucovorin). This combination induced a significantly higher percentage of apoptosis, secretion of HMGB-1, and expression of CALR and ERp57 than each therapy alone. Moreover, DCs were at a higher maturation state, and antigen presentation in non-draining lymph nodes and spleen was increased. Accordingly, at the tumour site, the presence of cytotoxic CD8+ T-cells was improved, and long-term immunological memory was demonstrated [56]. 

An alternative approach is to deliver cold atmospheric plasma-treated phosphate-buffered saline (pPBS), a gas composed of reactive oxygen and nitrogen species, as an ICD inducer. In a study by Van Loenhout et al. [57] conducted on cell line coculture of PCC and pancreatic stellate cells (PSC), the release of HMGB-1 was boosted in PCC lines, and treated cells were phagocytosed by DCs more efficiently than untreated cells. In fact, DCs expressed markers of activation, such as CD86, probably induced by the increased production of TNF-α and IFN-γ, which are known essential cytokines for DCs maturation. These results demonstrate that the treatment with pPBS can induce ICD in PCCs and render the TME more immunogenic. In vivo models are needed to determine whether the combination of this strategy with immunotherapy could have an impact on the disease course.

A different approach to induce immunogenicity is to mimic a viral infection through the production of type I interferon. Retinoic acid-inducible gene I-like helicases (RLH), a cytosolic helicase that induces an antiviral response, has been exploited as a therapeutic target in PDAC murine models. Treatment with RLH ligands induced the production of pro-inflammatory cytokines, such as type I IFN, and the subsequent immunogenic response via the activation of DCs. PCCs treated with RLH-ligands expressed ICD markers (CALR, HMGB-1, and HSP70) [58].

Lastly, the intratumor injection of the TLR9 agonist IMO-2125 was reported to induce immunogenicity, with an effect similar to ICD induction, culminating in CD8+ T-cell activation and response against tumours. TLR9 is expressed by several TME components, and the activity of IMO-2125 was previously demonstrated in colorectal and pancreatic cancer in vivo models. A study by our group, conducted on PDAC mouse models, demonstrated that IMO-2125 administered alone or in combination with an ICI could inhibit tumour growth and stimulate the immune response [59]. In particular, the combination of IMO-2125 with ICI in the immunogenic subtype of PDAC achieved a higher rate of response both locally at the injected site and at distant untreated sites due to the activation of cytotoxic T-cells. Therefore, this treatment will be investigated in clinical practice in an upcoming phase I/II clinical trial. Moreover, these results suggest that subtyping PDAC could have important therapeutical implications, as IMO-2125 intratumoral injection in the low immunogenic potential subtype was less efficient at controlling local tumour growth and ineffective at distant sites, and in the subtype without immunogenic potential, no tumour growth inhibition was observed.

Similarly, the interim results of the NANT Cancer Vaccine phase II trial (NCT04390399) were presented at the 2022 annual ASCO meeting [60]. In this study, refractory PDAC patients were treated with a combination of DAMP-inducing chemotherapy (nab-paclitaxel, gemcitabine, aldoxorubicin, and cyclophosphamide), low-dose SBRT, L-15 cytokine fusion protein (N-803), and PDL-1-targeted high-affinity NK cell (PDL1-thNK) infusion. Safety and efficacy data were interesting, achieving an OS of 6.3 months in third-line patients, while the final results are awaited.

Overall, the induction of ICD seems interesting in PDAC, especially as a combination strategy with other chemo/immune/radiotherapy; these data need to be validated in the clinical setting.

## 6. ICD in Liver Cancer

The most common type of primary liver cancer is hepatocellular carcinoma (HCC), which frequently presents at an advanced stage and has limited therapeutic options. 

Patients with an unresectable HCC, in the absence of metastatic disease, are usually treated with locoregional palliative treatment, such as transarterial chemoembolisation (TACE), which is known to induce a T-cell response against tumour cells. To describe the composition of the TME of HCC after TACE, tumour samples from patients who received TACE and subsequent liver resection or transplantation were compared to those from patients who did not undergo TACE [61]. Both cytotoxic CD8+ T-cells and Tregs were found to be lower in TACE-pre-treated samples. Even if cytotoxic T-cells do not increase as expected with ICD, the depletion of Treg cells is relevant, as it could be used as a therapeutic option in combination with ICIs. This strategy was investigated in a randomised phase II clinical trial (NCT03572582), in which patients received up to two TACE followed by treatment with nivolumab and achieved an overall response rate (ORR) of 71.4%, without safety concerns [62]. According to the principle of potentiating T-cell response, the combination of TACE plus low-dose cyclophosphamide and DC vaccination showed an increased mPFS in respect to patients who did not receive the vaccination (18.6 vs. 10.4 months, HR 0.43) and a higher ORR (54% vs. 29%); thus, further investigation of DCs’ infusion is warranted [63]. In a phase II clinical trial, SBRT was combined with a PD-1 antibody, sintilimab, in patients with recurrent or oligometastatic disease, yielding an impressive ORR of 96%; however, survival data are not mature enough to draw conclusions [64]. 

Notably, in line with other cancer types, oxaliplatin was found to be an ICD inducer in both murine and human HCC cell lines. Indeed, murine cells treated with oxaliplatin showed increased expression of CALR, HMGB-1, and ATP, and the number of mature DCs improved. These results were confirmed in murine models. Interestingly, the combination of oxaliplatin with anti-PD-1 had a synergistic effect in inhibiting tumour growth and in inducing DCs maturation and CD8+ T-cells activation [65]. Nonetheless, oxaliplatin has no role in HCC treatment. 

## 7. Conclusions and Future Perspectives

In this review, we highlighted different mechanisms of ICD induction, mainly applied in preclinical models, to boost the immune system against cancer cells in colorectal, gastric, pancreatic and hepatocellular carcinomas. This concept is relatively recent, as it was defined in 2005 when doxorubicin was recognised as a cytotoxic agent able to elicit ICD in vitro, ex vivo, and in vivo [2]. Certain chemotherapeutic agents, known as ICD inducers, can increase tumour immunogenicity, and their possible combination with immunotherapy could be a strategy to overcome the immunosuppressive TME of GI cancers. This rationale seems appealing, given that the addition of ICIs to a chemotherapy backbone is usually tolerated well with a manageable safety profile, and oxaliplatin, a well-known ICD inducer, is already widely used in the standard treatment of colorectal, gastric, and pancreatic cancers and thus can be exploited in combination studies. Here, we reported some promising examples of ICIs in combination with ICD-inducers, such as the AtezoTRIBE or the AVANA trials in colorectal cancer or the DANTE and the PANDA trials in gastric cancer. Interestingly, ICD not only enhances the activation and recruitment of CD8+ cytotoxic T-cells and DCs at tumour sites but is also capable of providing immunological memory, therefore representing a potential vaccination against cancer. In this regard, PDAC could represent a challenging model, as novel therapeutic approaches in this setting are urgently needed, and the TME plays a major role. Due to the recent advances in basic research, in the near future, we expect further characterisation of the molecular pathways inducing DAMPs release and of ICD regulators in order to better define their therapeutical role. Gastrointestinal malignancies still have limited effective treatment options and are generally characterised by an immunosuppressive TME, representing an ideal scenario for validating this approach. Further studies on patients are needed to translate it into clinical practice and possibly exploit it as a mechanism of cancer immunosurveillance.

## Figures and Tables

**Table 1:** Mechanism of ICD induction for selected ICD inducers.

ICD Inducers	CALR Exposure	HMGB-1 Release	eIF2α Phosphorylation	ANXA1 Release	ATP Release	HPS70 Exposure	IFN-1 Release
Oxaliplatin	x	x	x		x	x	x
Anthracyclines	x	x	x	x	x		x
Bleomycin	x	x	x		x		
Radiotherapy	x	x			x		x
Photodynamic therapy	x	x			x		
Oncolytic virotherapy	x	x			x		x
Extracorporeal phototherapy	x	x			x		

ICD—immunogenic cell death; CALR—calreticulin; HMGB-1—high-mobility group box-1; eIF2α—eukaryotic translation initiation factor 2 subunit α; ANXA1—annexin A1; ATP—adenosine triphosphate; HPS70—heat shock protein 70; IFN—interferon.

## Data Availability

Not applicable.
